# Reduced Basal ATP Synthetic Flux of Skeletal Muscle in Patients with Previous Acromegaly

**DOI:** 10.1371/journal.pone.0003958

**Published:** 2008-12-18

**Authors:** Julia Szendroedi, Elisabeth Zwettler, Albrecht Ingo Schmid, Marek Chmelik, Giovanni Pacini, Gertrud Kacerovsky, Gerhard Smekal, Peter Nowotny, Oswald Wagner, Christoph Schnack, Guntram Schernthaner, Klaus Klaushofer, Michael Roden

**Affiliations:** 1 1st Med. Department and Karl-Landsteiner Institute for Endocrinology and Metabolism, Hanusch Hospital, Vienna, Austria; 2 Institute for Clinical Diabetology, German Diabetes Center, Leibniz Center for Diabetes Research, Düsseldorf, Germany; 3 4th Med. Department and Ludwig Boltzmann Institute of Osteology, Hanusch Hospital, Vienna, Austria; 4 MR Centre of Excellence, Med. University Vienna, Vienna, Austria and Karl-Landsteiner Institute for Endocrinology and Metabolism, Hanusch Hospital, Vienna, Austria; 5 National Research Council, Padova, Italy; 6 Institute of Sports Sciences, Department of Sport Physiology, University Vienna, Vienna, Austria; 7 Department of Internal Medicine 3, Medical University of Vienna, Vienna, Austria; 8 Department of Medical and Chemical Laboratory Diagnostics, Medical University of Vienna, Vienna, Austria; 9 Med. 1, Rudolfstiftung Hospital, Vienna, Austria; 10 Department of Medicine/Metabolic Diseases, Heinrich Heine University Düsseldorf, Düsseldorf, Germany; University of Las Palmas de Gran Canaria, Spain

## Abstract

**Background:**

Impaired mitochondrial function and ectopic lipid deposition in skeletal muscle and liver have been linked to decreased insulin sensitivity. As growth hormone (GH) excess can reduce insulin sensitivity, we examined the impact of previous acromegaly (AM) on glucose metabolism, lipid storage and muscular ATP turnover.

**Participants and Methods:**

Seven AM (4f/3 m, age: 46±4 years, BMI: 28±1 kg/m^2^) and healthy volunteers (CON: 3f/4 m, 43±4 years, 26±2 kg/m^2^) matched for age and body mass underwent oral glucose testing for assessment of insulin sensitivity (OGIS) and ß-cell function (adaptation index, ADAP). Whole body oxidative capacity was measured with indirect calorimetry and spiroergometry. Unidirectional ATP synthetic flux (fATP) was assessed from ^31^P magnetic resonance spectroscopy (MRS) of calf muscle. Lipid contents of tibialis anterior (IMCLt) and soleus muscles (IMCLs) and liver (HCL) were measured with ^1^H MRS.

**Results:**

Despite comparable GH, insulin-like growth factor-1 (IGF-I) and insulin sensitivity, AM had ∼85% lower ADAP (p<0.01) and ∼21% reduced VO_2_max (p<0.05). fATP was similarly ∼25% lower in AM (p<0.05) and related positively to ADAP (r = 0.744, p<0.01), but negatively to BMI (r = −0.582, p<0.05). AM had ∼3fold higher HCL (p<0.05) while IMCLt and IMCLs did not differ between the groups.

**Conclusions:**

Humans with a history of acromegaly exhibit reduced insulin secretion, muscular ATP synthesis and oxidative capacity but elevated liver fat content. This suggests that alterations in ß-cell function and myocellular ATP production may persist despite normalization of GH secretion after successful treatment of acromegaly.

## Introduction

Acromegaly generally results from slowly growing monoclonal pituitary adenomas secreting growth hormone (GH). As a result of its slow and often insidious onset, it frequently remains unrecognized for an extended time, which may give rise to sustained metabolic alterations [1]. Although improving, the overall standardized mortality of patients with acromegaly is about 1.5fold higher compared with the general population [2], [3]. Among other factors, the higher prevalence of glucose intolerance and overt diabetes mellitus could contribute to the increased mortality [4].

GH stimulates protein anabolism and at the same time augments lipolysis and reduces insulin-dependent glucose disposal [5]. Patients with active acromegaly frequently exhibit mild hepatic [6] and more pronounced muscular insulin resistance [7], [8] which is similar to the metabolic alterations during aging and states of obesity, inherited risk of or overt type 2 diabetes (T2DM). Noninvasive magnetic resonance spectroscopy (MRS) made it possible to identify impaired muscle glucose transport/phosphorylation from monitoring basal and insulin stimulated glucose-6-phosphate (G6P) concentrations as well as reduced hepatic glycogen synthesis as key mechanisms underlying reduced glucose disposal in human insulin resistance [9]. According to the current paradigm, inherited and environmental factors (elevated glucose or free fatty acids, FFA) lead to reduced Krebs cycle flux and ATP synthetic flux (fATP) suggesting impaired mitochondrial oxidation/phosphorylation which in turn increases the ectopic deposition of lipids in hepatocytes (hepatocellular lipids, HCL) and myocytes (intramyocellular lipids, IMCL) [10]. Mitochondrial function is impaired in insulin resistant states including obese nondiabetic humans, relatives of patients with T2DM and overt T2DM. Electron microscopic examination revealed abnormalities of the morphology of muscle mitochondria in a patient with acromegaly which resolved upon surgical treatment [11]. Recently, evidence was provided that an acute 4-fold increase in plasma GH not only increased plasma insulin-like growth factor-1 (IGF-I), insulin, glucose and FFA but also shifted fuel selection into the direction of fat oxidation and stimulated muscle ATP production rate and citrate synthase activity [12]. However, to our knowledge, no data are currently available on muscle ATP synthesis in patients with acromegaly.

In addition, patients with active acromegaly may also feature altered ß cell function with hyperinsulinemia either resulting from direct effects of GH/IGF-I or from adaptation to changes in glucose and FFA availability [13], [14]. Glucose intolerance secondary to acromegaly generally improves following treatment of the underlying disease, whereas no data are available on ATP synthesis and ectopic lipid deposition under these conditions.

We tested the hypothesis that the alterations associated with active acromegaly are completely reversed by normalizing the endocrine and metabolic environment implicating normal fATP. To this end, we measured insulin sensitivity and ß cell function as well as whole body energy expenditure and physical fitness. Employing noninvasive multinuclear magnetic resonance spectroscopy (MRS), we further assessed in vivo fATP, flux through creatine kinase (fCK) and glucose metabolites (G6P) in skeletal muscle as well as ectopic lipid deposition (IMCL, HCL) in patients with prior acromegaly.

## Methods

### Volunteers

We included seven patients with prior acromegaly (AM) and seven healthy subjects (CON) matched for age, BMI and physical activity [15]. The mean duration between assumed clinical onset based on typical symptoms and diagnosis of acromegaly was 10±4 years. The participants had no first-degree relatives with T2DM. The mean interval between successful treatment until inclusion into the study was 14±3 years, but at least 7 years. All but one patient underwent transsphenoidal surgery for GH-secreting pituitary adenomas, one patient also received adjuvant radiotherapy and gamma knife treatment. Two of them required replacement of hormones (thyroid hormone, hydrocortisone and in one case additionally estrogen and gestagens) due to postoperative hypophyseal insufficiency. Three patients had been on pre- and postsurgical treatment with somatostatin analogues and bromocriptin, but only one patient required pharmacological treatment of acromegaly during the last two years. This patient was on octreotide and pegvisomant, had normal GH and IGF-I concentrations and similar insulin secretion compared to the rest of the group, but was glucose intolerant based on the 2-h plasma glucose concentration during the oGTT. Fasting plasma concentrations of IGF-I and GH and suppression during OGTT were within the normal range at repeated measurements during the last years indicating successful treatment in patients with acromegaly.

### Experimental protocol

The protocol was approved by the local human ethical board (Ethics Committee of the Medical University of Vienna), and written informed consent was obtained from each volunteer. All participants were instructed to ingest a mixed diet (25–30 kcal per kg bodyweight per day consisting of 60% carbohydrates, 25% fat, 15% proteins) for three days prior to the studies. They refrained from any physical exercise for three days and fasted for 12 hours and did not take any medications before the experiments. A 75-gram oral glucose tolerance test (OGTT) was performed to assess glucose tolerance, insulin sensitivity and secretion. Magnetic resonance spectroscopy (MRS) and respiratory gas exchange measurements were carried out on separate days with intervals of at least one week each at identical daytimes in the morning.

### MRS

All measurements were performed during resting conditions in participants lying supine inside a 3-T spectrometer (Bruker, Germany) using a 10-cm circular double resonant surface coil for ^1^H/^31^P measurements [16]. ^31^P MRS allowed determination of fATP from the exchange between inorganic phosphate (Pi) and ATP (Pi→ATP) applying saturation transfer to calf muscle as described [16], [17]. In analogous fashion, the exchange between phosphocreatine (PCr) and ATP (PCr→ATP) was used to calculate total (cytosolic and mitochondrial) flux through creatine kinases (fCK) [18], [19]. Intramyocellular concentrations of G6P, Pi, PCr and phosphodiesters (PDE) were measured from the ratio of the respective integrated peak intensities and ß-ATP resonance intensity in spectra without inversion and saturation assuming constant ATP concentrations of 5.5 mmol/l muscle [20]. ^1^H MRS allowed to measure IMCL in soleus and tibialis muscles [21] and HCL as described [22].

### OGTT

The volunteers drank a solution containing 75 grams of glucose and venous blood samples were collected before and in 30-min intervals during 150 min for measurements of plasma glucose, insulin, C-peptide. Plasma concentrations of insulin, C-peptide and IGF-I were determined by double antibody radio immunoassay [22]. Further, dynamic insulin sensitivity were assessed with the OGIS [23], a measure of glucose clearance which has been validated against insulin sensitivity obtained from the euglycemic-hyperinsulinemic clamp [24]. Insulin secretion in relation to ambient insulin sensitivity was assessed with the insulinogenic index (ISEC), an indicator of first phase insulin secretion which is calculated as the ratio between the supra-basal increments at 30 min of insulin and glucose concentration [24]. The adaptation index (ADAP) is based on the areas under the concentration curves of plasma glucose and C-peptide during the OGTT and is a marker of responsive ß cell function [25].

### Respiratory gas exchange measurement

After a 30-min resting period, measurements were performed on subjects in supine position under a canopy using an open-air spirometry system (Jaeger/Viasys MasterScreen CPX. Wuerzburg. Germany) combined with continuous heart rate recording (Sporttester PE4000, Polar Electro, Kempele, Finland). Resting energy expenditure (REE, kcal/24 h) was assessed from two 30-min sets which were separated by 15-min breaks in a quiet air conditioned laboratory (21°C) using the equation: 3.91 VO_2_ −1.10 VCO_2_ – 1.93 N [26], [27]. Thereafter, the volunteers performed an incremental exercise test on an electronically-braked cycle ergometer (Lode Excalibur Sport, Groningen, Netherlands). The exercise test started at an initial level of 20 W followed by 7, 10, 15 or 20 W increments every min until exhaustion. The respiratory compensation point (RCP) was determined from incremental testing as described previously [28].

### Statistical analysis

Data are presented as means±SEM. Statistical comparisons between study groups were performed using two-tailed Student's *t* tests or the Mann-Whitney-Wilcoxon test for parameters not having a normal distribution (ADAP, ISEC, GH, IGF-I, HCL, IMCLs) as assessed from the Levene test. Linear correlations are Pearson product-moment correlations. Differences were considered significant at the 5% level.

## Results

**Figure 1:**
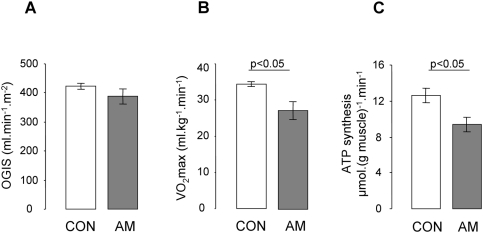
Whole-body insulin sensitivity, maximal oxygen consumption and muscle mitochondrial ATP production (means±SEM): (A) OGIS (B) VO_2_max (p<0.05) and (C) flux through ATP synthesis (fATP) (p<0.05) in 7 subjects with previous acromegaly (AM, full bars) and 7 age- and body mass index-matched controls (CON, empty bars).

Anthropometric and laboratory data are summarized in **Table 1**. In AM, fasting plasma concentrations of IGF-I and GH did not differ from CON and were within the normal range indicating successful treatment of the patients with previous acromegaly. Fasting plasma glucose was ∼17% higher in AM (p<0.05) and four patients had impaired fasting glucose (defined as fasting plasma glucose levels of 5.6 to 6.9 mmol/l), whereas plasma insulin and C-peptide were not different. All but one participant had normal glucose tolerance (as defined by the 2-h plasma glucose concentration less than 7.8 mmol/l the during OGTT) during active acromegaly and at follow-up visits following successful treatment. Fasting plasma insulin and C-peptide related positively to IGF-I levels (r = 0.552 and r = 0.646, p<0.05). Insulin secretion indices (ADAP, ISEC) were ∼85% lower in AM than in CON (p<0.05). Insulin sensitivity (OGIS) was comparable between groups (**Figure 1**) and correlated negatively with fasting plasma FFA (r = −0.744, p<0.05). FFA further related positively to GH levels (r = 0.782, p<0.01).

**Table 1:** Anthropometric and laboratory data (means±SEM) in subjects with prior acromegaly (AM) and controls (CON).

	AM	CON
**N (f/m)**	4/3	3/4
**Age (years)**	46±4	43±4
**BMI (kg/m^2^)**	27.8±1.3	25.6±1.5
**Waist-to-hip ratio**	0.86±0.02	0.86±0.04
**HbA1c (%)**	5.4±0.2	5.3±0.1
**Insulin-like growth factor-1 (IGF-I, ng/ml)**	227±59	141±18
**Growth hormone (GH, U/ml)**	2.4±0.6	3.3±1.5
**Fasting glucose (mmol/l)**	5.5±0.2	4.7±0.1 *
**2-hour glucose (mmol/l)**	6.2±0.9	5.2±0.4
**Fasting insulin (pmol/l)**	43.4±8.2	37.2±5.7
**Fasting C-peptide (pmol/ml)**	2.4±0.3	1.9±0.3
**Fasting plasma FFA (μmol/l)**	368±96	285±29
**ISEC (×10^−6^)**	0.8±0.3	6.5±1.5 §
**ADAP (l.min^−1^.m^−2^.10^−3^)**	0.4±0.2	2.7±0.6 §

* P<0.01.

§ P<0.05 vs. AM.

Parameters of whole body oxidative capacity during resting and exercise are summarized in **Table 2**. REE, resting O_2_ consumption (AM: 0.25±0.02, CON: 0.28±0.02 l.min^−1^) and CO_2_ production (AM: 0.20±0.01, CON: 0.23±0.02 l.min^−1^), the respiratory quotient (RQ), as well as the derived oxidation rates for glucose (GOX) lipids (LOX) and proteins (POX) were comparable. GOX related negatively to FFA (r = −0.718, p<0.05) and GH (r = −0.729, p<0.05) and LOX related positively to skeletal muscle fATP (r = 0.637, p<0.05).

**Table 2:** Expired gas analysis during resting (indirect calorimetry) and exercise (spiroergometry).

	AM	CON
**Resting energy expenditure (kcal/24 h)**	1678±118	1889±167
**Glucose oxidation (mg.kg^−1^.min^−1^)**	1.5±0.2	1.4±0.3
**Lipid oxidation (mg.kg^−1^.min^−1^)**	0.6±0.1	0.7±0.1
**Protein oxidation (mg.kg^−1^.min^−1^)**	0.7±0.1	0.7±0.2
**Respiratory quotient**	0.84±0.04	0.83±0.04
**VO_2_max (ml.kg^−1^.min^−1^)**	27±2	34±1 §
**Maximal power output (W)**	180±26	229±17
**VO_2_rcp (ml.kg^−1^.min^−1^)**	22±2	28±1 §
**Power output at RCP (W)**	130±16	179±12

Fasting substrate oxidation and parameters of physical fitness (means±SEM) in subjects with prior acromegaly (AM) and controls (CON).

§ P<0.05 vs. AM.

AM had ∼21% lower maximal oxygen uptake (VO_2_max) and oxygen uptake at the RCP (VO_2_rcp) (p<0.05, **Figure 1**), the respective power output measures tended to be lower in AM but did not reach significance.

Intramyocellular G-6-P (both groups: 0.09±0.01 mmol/L) and pH (7.05±0.0) were comparable in both groups. Skeletal muscle fATP was ∼25% lower in AM (p<0.05, **Figure 1**) and related positively to ADAP (r = 0.744, p<0.01) and ISEC (r = 0.687, p<0.01) and negatively to BMI (r = −0.582, p<0.05). The rate constant of ATP synthesis (*k*1) was ∼25% lower in AM (AM: 0.06±0.01, CON: 0.08±0.01 s^−1^, p<0.01), PCr/Pi ratios (AM: 7.7±0.6, CON: 7.3±0.2) and fCK (AM: 317±23, CON: 350±13 µmol.l^−1^.min^−1^) were comparable.

**Figure 2:**
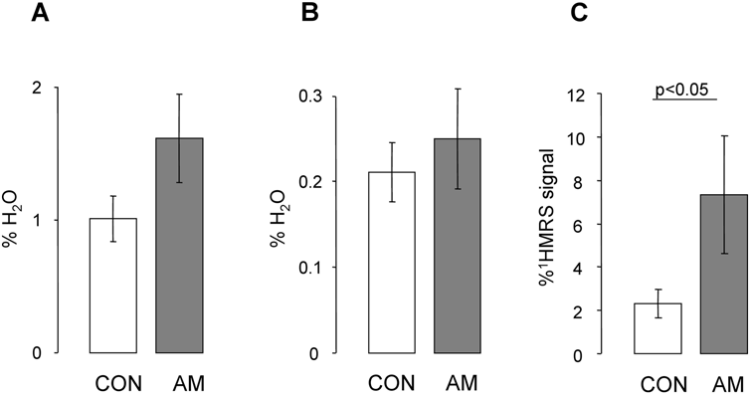
Ectopic lipid deposition: (A) in M.soleus (IMCLs), (B) M.tibialis ant. (IMCLt) and (C) in the liver (HCL) (p<0.05).

**Figure 3:**
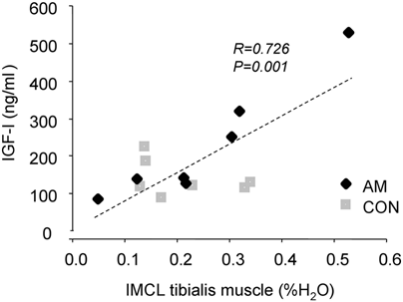
Relationship between ectopic lipid content of tibialis muscle (IMCLt) and plasma concentrations of insulin-like growth factor-1 (IGF-I) in 7 subjects with previous acromegaly (AM, black squares) and 7 age- and body mass index-matched controls (CON, grey squares) (r = 0.726, p<0.05).

HCL was three-fold higher in AM than in CON (**Figure 2**). IMCLs and IMCLt did not differ between the groups. IMCLs, but not IMCLt, related negatively to insulin sensitivity (r = −0.799, p<0.001). IMCLt, but not IMCLs, related positively to plasma IGF-I (r = 0.726, p<0.01) (**Figure 3**).

## Discussion

Humans with a history of acromegaly exhibit normal whole body insulin sensitivity despite impaired ß cell function. Nonetheless, noninvasive measurements unmasked abnormalities in energy metabolism such as impaired muscular ATP synthesis during resting conditions, reduced maximal oxygen consumption and increased hepatic triglyceride contents years after successful treatment of acromegaly. This is similar to the reduced mitochondrial function observed in elderly or type 2 diabetic patients [17], [29], [30].

This study is consistent with previous reports showing that whole-body insulin sensitivity is normal after cure of acromegaly [31], [32]. It is well known that reduced insulin-sensitive muscular glucose uptake in untreated acromegalic patients is mediated by prolonged increases in GH and IGF-I [8], [33]. The cellular mechanisms of acromegaly-associated insulin resistance involve direct GH-mediated expression of suppressors of cytokine signaling (SOCS-1/-6) [34] or indirect activation of nutrient-sensing pathways [35] due to excessive lipolysis [20] and proteolysis [36], [37]. Dietary proteins and amino acids promote insulin resistance via the mammalian target of rapamycin, a nutrient sensor that activates a negative feedback loop toward insulin receptor substrate-1 signalling [37]. But for the most part, increased plasma FFA can cause serine phosphorylation of insulin-receptors substrate-1 and thereby inhibit proximal steps of insulin signaling [38]. Accordingly, in the present study, FFA related positively to plasma levels of GH and negatively to insulin sensitivity. In addition, therapeutic interventions may also affect insulin action and secretion: first, transsphenoidal surgery can either be not sufficient to normalize GH and IGF-I secretion or alternatively lead to deficiency of other hypophyseal hormones involved in metabolism [39] and second, somatostatin analogues and GH receptor analogues can specifically affect insulin secretion or glucose metabolism. In the present study, only one patient was on acromegaly-specific medication, but all patients had plasma concentrations of GH, IGF-I, other hypophyseal hormones as well as circulating FFA which were not different from the matched control group. Thus, it is not surprising that whole-body insulin sensitivity was not impaired in these patients with a history of acromegaly.

On the other hand, the participants with previous acromegaly exhibited severe impairment of dynamic insulin secretion. Both insulin and IGF-I are important regulators of ß cell development, ß cell mass and function [40], [41]. Chronic GH/IGF-I excess during active acromegaly induces hyperinsulinemia which is likely mediated by elevation of plasma glucose as well as by direct ß cytotrophic effects [13], [41]. Accordingly, IGF-I levels related positively to fasting plasma concentrations of insulin and C-peptide in the present study. During early stages of T2DM, exhaustive stimulation of ß cells to counterbalance insulin resistance precedes the progressive ß cell failure involving impaired function and loss of ß cell mass [42]. The pathophysiological mechanisms might involve excess of nutrient availability, particularly of FFA [43], hyperglycemia, adipocytokine-mediated endoplasmic reticulum stress (release of reactive oxygen species) and cellular inflammation [44]. These mechanisms could also apply for the present study, assuming that the participants acquired their ß cell defect during active acromegaly. Ultimately, insulin secretory responses rely on intact mitochondrial function to provide for sufficient ATP and - as recently demonstrated - GTP for glucose-stimulated insulin secretion [45]. Thus, mitochondrial impairment of ß cells could also underly reduced adaptive insulin secretion.

During active acromegaly, there is evidence that GH acutely reduces glucose oxidation and impairs glucose disposal directing glucose fluxes into the non-oxidative pathway. In line with this contention, rates of lipid oxidation and non-oxidative glucose metabolism were found to be increased while oxidative glucose metabolism was decreased [31], [46]. However, this seems to hold true for high plasma FFA concentrations because no reduction in oxidative metabolism was detected in the presence of normal plasma FFA [8], [47]. Accordingly, in the present study GOX related negatively to FFA and GH. However, resting energy expenditure, as well as substrate specific oxidation rates did not differ between the study groups. On the other hand, despite comparable physical activity oxygen uptake during maximal exhaustion and at the RCP were reduced in the group of patients with previous acromegaly. The RCP marks the onset of hyperventilation during incremental exercise mainly driven by the onset of lactic acidosis [48] and thus is a good marker of oxidative capacity.

To our knowledge, no previous data are available on in vivo ATP production in humans with prior acromegaly. Here, such humans showed reduction of ATP synthetic flux rates (fATP) to a similar extent as reported in other states of impaired oxidative phosphorylation [30], [49]. This is also reflected by the positive relationship between fasting whole body lipid oxidation and skeletal muscle fATP. It has been shown that in combination with endurance training, GH injections can enhance the positive effects of physical activity on muscle mitochondrial enzyme activities [50]. A recent report also investigated the sole effects of continuous GH infusion for 14 h and found that GH not only induced several mitochondrial genes but also promoted an increase in mitochondrial capacity in particular for fat oxidation and a shift in whole body fuel utilization toward enhanced fat utilisation [12]. Augmented lipid availability leads to accumulation of lipid metabolites such as long-chain fatty acyl CoA, diacylglycerols and ceramides which are known to impair insulin signalling and damage mitochondria [10]. In the present study, insulin sensitivity related negatively to IMCL of soleus muscle, although IMCL were comparable in both groups. Nevertheless, the relationship between IMCL of tibialis muscle and IGF-I suggests that active acromegaly could favour prolonged lipid accumulation with long-term deleterious effects on mitochondria. On the other hand, patients with prior acromegaly had markedly increased HCL. In the absence of alcohol intake or other hepatotoxic agents, HCL accumulation, i.e. steatosis or non-alcoholic fatty liver [51], could be a key factor in the development of insulin resistance and T2DM [52]. These data are in line with a previous study reporting 4–7fold higher HCL, but unchanged IMCL contents in T2DM with impaired fATP [30]. Thus, excessive hepatic lipid storage relates to insulin resistance, hepatocellular mitochondrial dysmorphology, depletion of mtDNA and decreased activity of ETC and could be interpreted as mitochondrial impairment of hepatocytes [53].

Of note, one limitation resides in the fact that the study design does not allow discrimination whether these abnormalities directly result from the previous GH/IGF-I excess or develop as a long-term consequence of metabolic alterations due to glucose, lipids and adipocytokines, because no patients with active acromegaly were included in this study. It is also impossible to sort out whether dysfunction of ß cells or of myocytes occurs first and to which extent these alterations are interrelated. Furthermore, the observed reduction in basal ATP synthesis flux does not equal mitochondrial dysfunction but could reflect a decreased ATP demand, a decreased mitochondrial content, and/or an impaired intrinsic mitochondrial function [54].

Long-term prospective studies in patients before and after successful acromegaly treatment are required to address these issues. Regardless of the cause and sequence of abnormalities in humans with prior acromegaly, the sustained disruption of ATP synthesis along with severely impaired ß cell function could contribute to an increased risk of this population for developing diabetes despite successful acromegaly therapy.
